# Patient-specific factors influence somatic variation patterns in von Hippel–Lindau disease renal tumours

**DOI:** 10.1038/ncomms11588

**Published:** 2016-05-13

**Authors:** Suzanne S. Fei, Asia D. Mitchell, Michael B. Heskett, Cathy D. Vocke, Christopher J. Ricketts, Myron Peto, Nicholas J. Wang, Kemal Sönmez, W. Marston Linehan, Paul T. Spellman

**Affiliations:** 1 Department of Molecular & Medical Genetics, Oregon Health & Science University, Mail Code: CL6S, 2730 SW Moody St, Portland, Oregon 97201, USA; 2 Urologic Oncology Branch, Center for Cancer Research, National Cancer Institute, Building 10 Room 1-5940, Bethesda, Maryland 20892, USA; 3 Department of Biomedical Engineering, Oregon Health & Science University, Mail Code: CH13B, Portland, Oregon 97201, USA

## Abstract

Cancer development is presumed to be an evolutionary process that is influenced by genetic background and environment. In laboratory animals, genetics and environment are variables that can largely be held constant. In humans, it is possible to compare independent tumours that have developed in the same patient, effectively constraining genetic and environmental variation and leaving only stochastic processes. Patients affected with von Hippel–Lindau disease are at risk of developing multiple independent clear cell renal carcinomas. Here we perform whole-genome sequencing on 40 tumours from six von Hippel-Lindau patients. We confirm that the tumours are clonally independent, having distinct somatic single-nucleotide variants. Although tumours from the same patient show many differences, within-patient patterns are discernible. Single-nucleotide substitution type rates are significantly different between patients and show biases in trinucleotide mutation context. We also observe biases in chromosome copy number aberrations. These results show that genetic background and/or environment can influence the types of mutations that occur.

## Abstract (web summary)

Analysing multiple tumours from the same patient permits the study of the germline contribution to cancer. Here, the authors sequence multiple renal tumours from VHL patients and find that intra-patient tumours are clonally distinct but share some genetic features, suggesting that patient-specific factors influence tumour formation.

## Graphical abstract

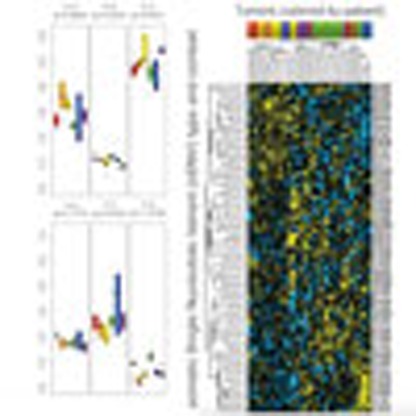

Cancer development, like evolution, is thought to be largely stochastic. Somatic variants occur randomly, and if they provide a fitness advantage, the cells with the mutations expand clonally^1^. The ‘hallmarks of cancer' proposition hypothesizes that many biological processes are typically mutated or dysregulated in cancer^2^,^3^. As somatic variation accumulates in the cells, the likelihood that all of the necessary pathways are affected increases and, when the conditions are right, cancer develops.

Although somatic variation occurs stochastically, the types of variants and whether they are selectively advantageous are often influenced by both the genetic background and environment of the patient. For example, tumours from patients with germline BRCA mutations and tumours from patients who smoke have characteristic single-nucleotide variant patterns^4^. It is unknown how much cancer development is stochastic versus driven by genetic and environmental factors. It is possible to observe the stochasticity by holding the genetic background and environment constant, as can be done with laboratory animals. With humans, one approach for controlling for both genetic background and environmental effects is to observe independent tumours that develop within the same patient. This can occur when a patient inherits or has a *de novo* germline mutation in a cancer risk gene.

*VHL* is a tumour suppressor gene inactivated in clear cell renal cell carcinoma (ccRCC). *VHL* is located on chromosome 3p and is inactivated by mutation or methylation combined with chromosome arm loss in an estimated 90% of ccRCC tumours^5^,^6^. Families affected with von Hippel–Lindau disease carry germline mutations in *VHL*^7^. Patients with VHL disease are at risk to develop hundreds of independent kidney tumours and cysts during their lifetime. Long-term studies have shown that if surgical intervention is performed when the largest renal tumour reaches a threshold size of 3 cm, risk of metastasis is effectively zero^8^. Thus, patients with mutations in *VHL* are managed by active surveillance until the largest lesion reaches 3 cm, at which time surgical intervention is recommended. When possible, all identifiable tumours are removed from the kidney while sparing the maximum amount of normal kidney to preserve renal function^9^. This provides the opportunity to evaluate independent tumours of differing sizes and stages which are acquired at the same time point. Unlike sporadic ccRCC tumours that are large and genetically heterogeneous due to late diagnosis^10^,^11^, the tumours in VHL patients are typically closely monitored and removed at an early stage. This simplifies the process of identifying variants because most are clonal and tumours are relatively genetically homogenous^12^.

Distinct copy number break points in multiple ccRCC tumours from the same VHL patient provide strong evidence that these tumours arise independently^12^,^13^, and the sequencing of the exomes from four different tumours from the same patient showed no mutations in common which provided further evidence that the ccRCCs in VHL syndrome evolve independently^12^. Research from Fisher *et al*., found that all four of the VHL disease tumours had activated the PI3K/AKT/mTOR pathway providing tentative evidence that evolutionary constraints are operating, whereby earlier evolutionary history restricts later evolutionary events and in turn this can lead to convergent evolution^12^. Another example of similar constraints is found in immunosuppressed organ transplant patients who develop multiple independent, genetically distinct, squamous cell carcinomas. In these immunosuppressed individuals, copy number profiles show bias suggesting that factors within a patient influence the selection of oncogenic copy number events^14^. Further, in two cases of synchronous liver cancers similar mutations were observed^15^. Finally, the sequence of normal skin tissue has identified Notch gene family mutation bias between individuals^16^.

In most cancers, non-coding somatic single-nucleotide variants (sSNVs) vastly outnumber somatic copy number variants (sCNVs) and sSNVs found in coding regions of the genome. Typically there are more than 1,000 mutations in epithelial tumours in adult^17^,^18^. The larger amount of data provided by whole-genome sequencing allows for more detailed analysis of the somatically gained mutations present within each tumour, such as the rates of different nucleotide transitions and transversions, while also providing information on the copy number of the genome and the mutations within specific genes. Expanding on previous efforts in both scale of sequence and number of patients, we sequence the genomes of 40 tumours from six von Hippel–Lindau patients and perform a deep characterization of the mutation complement of tumours. We confirm that VHL tumours from the same patient are independent but that the combination of genetic and environmental background significantly influences both sCNVs and the types of sSNVs acquired.

## Results

### Samples and variant calling

The National Cancer Institute has a frozen tumour bank of ccRCC tumours that have been removed from VHL patients. We selected six patients, three males and three females, with at least five tumours from a single surgery from one kidney (Table 1 and Supplementary Data 1). They spanned a wide range of ages, body mass indices and smoking exposures. For three of the patients, adjacent normal kidney tissue was also available. A total of 42 tumours, 6 normal blood samples and 3 adjacent normal kidney tissue samples were sequenced using Illumina's Human Whole-Genome Sequencing service. The observed coverage distribution had a minimum of 29.3X, a maximum of 46.7X, a mean of 38.2X and a median of 39.0X. Median coverage for each sample is listed in Supplementary Data 1.

We identified sCNVs and sSNVs, respectively, in the tumours (Methods section). Other than where noted, somatic variant calls were made by comparing the DNA sequence of a tumour to the DNA sequence from white blood cells in the same patient. As expected, variant sets called by comparing tumour DNA sequence to normal kidney tissue DNA sequence were very similar to variant sets called by comparing tumour sequence to blood sequence. Two of the samples, one from patient M22red and one from patient M45orange, had few variants and clustered with the normal samples. Further inspection showed these samples were cyst-like rather than tumours, so they were excluded from further analysis.

**Figure 1:**
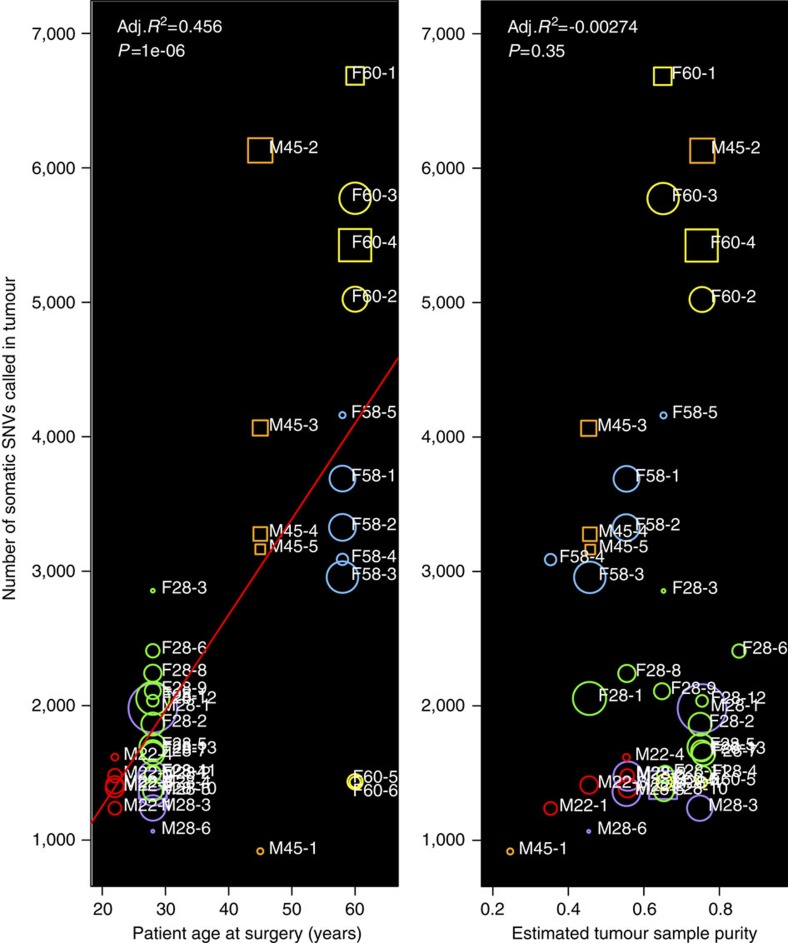
The number of somatic SNVs per tumour positively correlates with the patient's age at time of surgery. Colour indicates patient. Size indicates tumour size. Circles are Fuhrman grade 2, and squares are Fuhrman grade 3. The low outlier in patient M45orange was a small grade 2 tumour with lower purity, which could explain the reduced number of variants called. The low outliers in patient F60yellow, F60-5 and F60-6, both have low variant counts relative to the other tumours from that patient but neither were low purity.

In the remaining 40 tumours, we employed a three-step filtering process to produce a high-confidence list of sSNVs (Methods section). These variant call sets contained few false positives, as evidenced by the reduction in calls when comparing two normal samples from the same patient: before filtering: ∼4,000 calls; after sample-level filtering: ∼800 calls; after sample- and dataset-level filtering: ∼90 calls; after sample-, dataset- and pipeline-level filtering: ∼25 calls. The 100,677 high-confidence tumour versus blood sSNV calls were used for all subsequent analyses. The numbers of sSNVs per tumour ranged from 917 to 6,684 and were positively correlated with the age of the patient at time of surgery (Fig. 1 and Supplementary Data 1). There was no evidence of correlation between number of sSNVs and smoking exposure, body mass index or tumour sample purity.

### Comparison of variant sets

**Figure 2:**
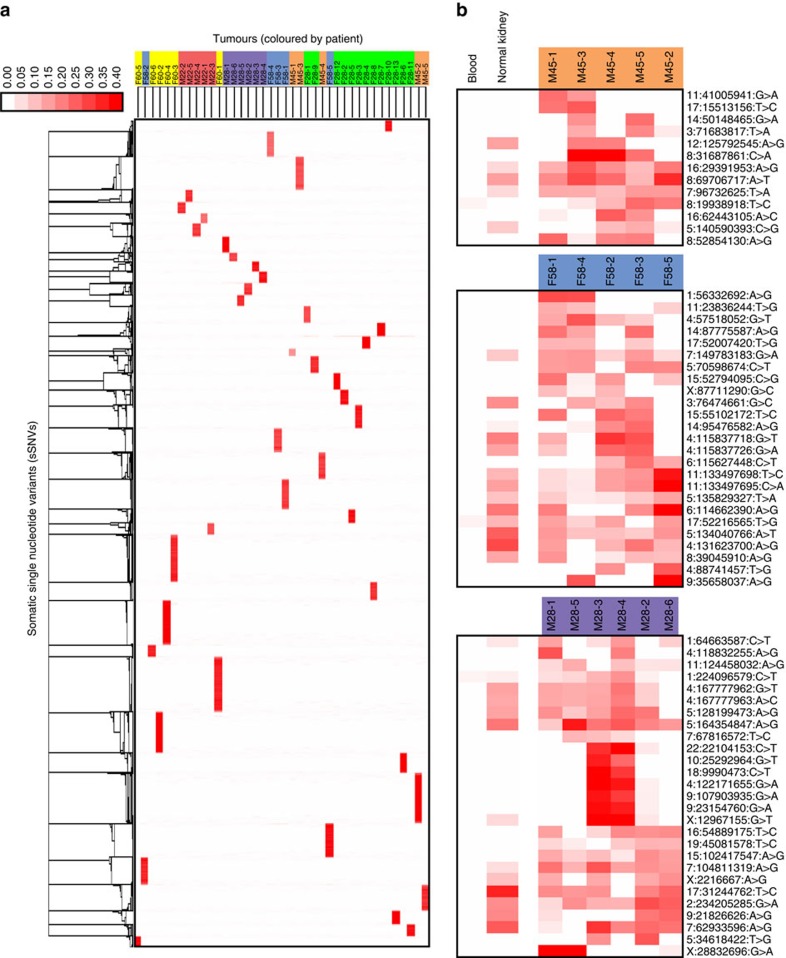
The somatic SNVs revealed that each tumour was independent from the other tumours. (**a**) 90.2% of the 100,677 variants called had sequencing reads found in only one tumour. Bright red indicates many reads matched the variant, and white indicates no reads matched variant. Lack of overlap in variant sets indicates the tumours are not clonally related. (**b**) A very small number of variants were called in more than one tumour from each patient. Patients M28purple, M45orange and F58blue had both blood and matched normal kidney tissue. Somatic variant calls were made by comparing tumour to blood; however, the presence of reads in the normal kidney tissue for variants called in more than one tumour is shown. Most of these variants were found in the normal kidney and not in the blood, suggesting they are actually kidney-specific rather than tumour-specific variants. M28-3 and M28-4 share seven variants not called in other tumours. Radiology confirmed these two tumours were adjacent to each other in the kidney.

Each tumour had a unique set of variants. Of all the called sSNVs, 90.2% had sequencing reads observed in only one tumour in a patient. If two tumours from the same patient originated from a single tumour, they would share a substantial set of variants. Significantly overlapping variant sets are absent, indicating the tumours arose independently (Fig. 2a). In the remaining 9.8% of sSNVs, reads matching the variant were found in two or more tumours from the same patient. For most of these sSNVs, however, the majority of the reads for each variant were found in a single tumour with the other tumour(s) from that patient containing only one or a few reads, thus the variants were almost always only called in one tumour (Fig. 2a).

A small number of variants had read evidence found in the majority of tumours from a single patient, and that number decreased considerably when dataset-level filtering was employed to remove potential germline variants and error-prone sites (Methods section). Only 138 (<0.2%) variants were called in more than one tumour from the same patient. Because we have both blood and normal kidney tissue for three patients, we were able to determine that half of the variants called in more than one tumour from those patients are likely kidney-specific variants that are absent in the blood (Fig. 2b).

### Comparison of variant types

**Figure 3:**
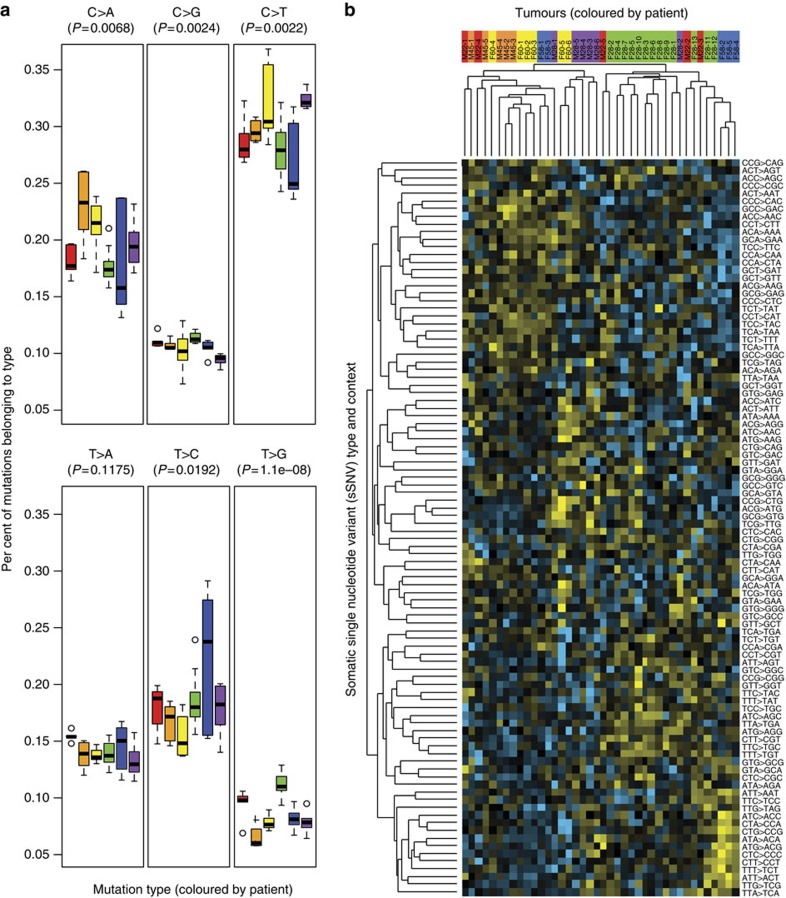
The genetic background and environment within a patient influence the types of SNVs that occur during tumour development. (**a**) There were significant differences between patients in the proportion of variants that belong to each variant type (ANOVA unadjusted *P*-values shown). Patient F28green in particular exhibited a significantly higher abundance of T>G variants compared with the other patients. Differences remain marginally significant when patient F28green is removed (ANOVA unadjusted *P*-values: C>A:0.094, C>G:0.13, C>T:0.011, T>A:0.13, T>C:0.038 and T>G:0.026). (**b**) Tumours from the same patient significantly cluster with each other when comparing the types of variants observed. The type of sSNV along with the base before and after it are shown. The raw data was a count of how often each variant type was seen in each sample. The rows and columns have been normalized and centred at zero to highlight differences between the tumours. Yellow indicates higher than average counts, and turquoise indicates lower than average counts. Tumours from the same patient had a higher average pairwise correlation than pairs selected at random (*P*=0.00014, Spearman rank correlation, 100,000 permutations). The likelihood of all of patient F28green's samples clustering on one half of the tree by chance is *P*=0.0000064 (hypergeometric distribution). Even when patient F28green is removed, pairwise correlations within the remaining patients still exceed those between random pairs (*P*=0.00241, Spearman rank correlation, 100,000 permutations). The default boxplot function in R was utilized where the box represents the 25th, 50th and 75th percentile; the upper whisker=min(max(x), Q_3+1.5 * interquartile range); and the lower whisker=max(min(x), Q_1–1.5 * interquartile range).

The number of whole-genome variants called permitted us to compare the types of sSNVs found in the tumours. The proportion of sSNVs belonging to each type was fairly consistent across tumours from a single patient; however, there were significant differences between patients, with the largest difference seen in T to G variants, which is the rarest type of sSNV. Patient F28green had a significantly higher proportion of T to G variants than the other patients (Fig. 3a). Patients F58blue and F60yellow show notable exceptions to the consistency of proportions within a patient. Several of their tumours show a wide range of proportions, perhaps due to early somatic changes having an influence on subsequent somatic variation. These two patients are also at least 13 years older than the next youngest patient in the data set.

To extend the basic sSNV type analysis shown in Fig. 3a, we also considered the variant's trinucleotide context: both the base before and the base after the sSNV as previously described^4^,^19^. After normalizing, centring at zero and performing clustering on both the rows and columns, clear differences between tumours emerged (Fig. 3b). Tumours from the same patient usually clustered together. This was especially clear for tumours from patient F28green due to the higher abundance of T to G variants. To determine if tumours from the same patient were more similar than tumours between patients, the averages of the pairwise Spearman rank correlations between tumours from the same patient were calculated. An average of the six patient averages was used to prevent unequal weighting of patients with more samples. Patient IDs were then randomly permuted and pairwise correlations were recalculated. Only 14 out of 100,000 random permutations had a higher average pairwise correlation, indicating that tumours from the same patient are significantly more similar than tumours from different patients.

At the time of writing, COSMIC contained 30 published signatures of mutational processes in cancer (http://cancer.sanger.ac.uk/cosmic/signatures)^4^,^20^,^21^,^22^,^23^. The signatures that were consistently the highest (Spearman *r*∼0.7) in the majority of our VHL tumours were: Sig1 (age-related), Sig5 (unknown aetiology) and Sig19 (unknown aetiology). Moderately high correlations (Spearman *r*∼0.6) were also consistently seen across patients with Sig2 (AID/APOBEC-related), Sig16 (unknown aetiology) and Sig27 (unknown aetiology but previously seen in ccRCC). The two tobacco-related signatures, Sig4 and Sig29, varied across patients (analysis of variance (ANOVA) *P*<0.00005), with Sig4 showing highest correlation with two tumours in M45orange, the patient with the highest estimated smoking exposure (Table 1). Sig29, however, also correlated with tumours in F60yellow and F58blue, patients who are nonsmokers. Mismatch repair-related signatures, Sig6, Sig15 and Sig20 also varied across patients (ANOVA *P*<0.0001). Their levels were very consistent within-patient, so small differences between patients led to significant *P* values. Like the single-nucleotide variant proportion results in Fig. 3a, the patient with the most signature variability within their tumours was F58blue. Her tumours showed a wide spread in correlations with a number of signatures, particularly Sig9 (unknown aetiology), Sig18 (unknown aetiology), Sig26 (mismatch repair-related) and Sig29 (tobacco-related, despite being a nonsmoker).

Four *de novo* mutational signatures were deciphered using SomaticSignatures^24^ and compared with the current set of mutational signatures listed by the Sanger Institute by cosine similarity. The *de novo* signatures were found to be most similar to COSMIC signature Sig3 (failure of double-strand break repair), Sig5 (unknown aetiology), Sig8 (unknown aetiology) and Sig16 (unknown aetiology); signatures that have not been previously associated with kidney cancer. This discordance is not surprising because COSMIC signatures were based primarily on exome sequencing. In addition, our mutation calls are the union set from multiple callers and are stringently filtered using multiple strategies resulting in a high-confidence call set. Significant differences in library preparation and analysis procedures make direct comparison to COSMIC signatures difficult.

### Comparison of copy number variation

**Figure 4:**
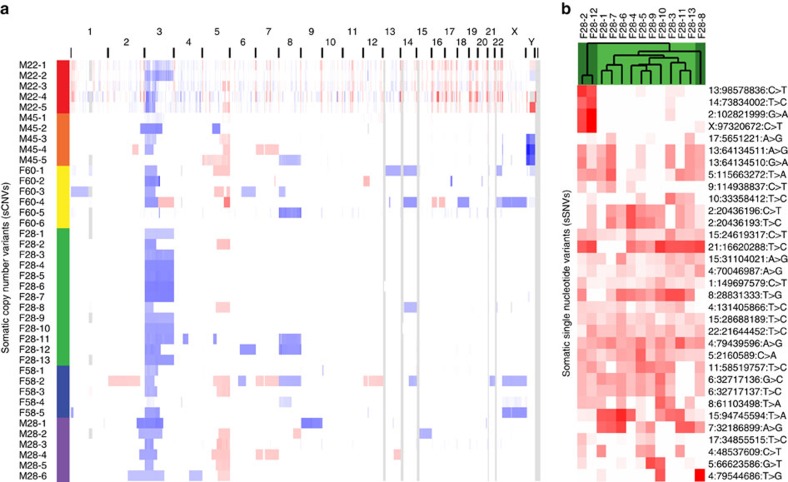
Copy number variants confirmed that the tumours are independent but showed within-patient patterns. (**a**) Copy number results: red bars indicate gains and blue bars indicate losses. The germline genetic background or environment in patient F28green predisposed her tumours to lose a complete copy of chromosome 3 over the more common loss of only 3p. Given that <10% of The Cancer Genome Atlas ccRCC tumours exhibited complete loss of chromosome 3, the likelihood of at least 10 out of 13 of patient F28green's tumours losing all of chromosome 3 by chance is 0.000000021 (binomial distribution). (**b**) Somatic SNVs in patient F28green: SNVs that were called in at least two tumours from patient F28green and had sufficient coverage in all tumours are shown. No single-nucleotide variants were called in the 10 tumours with chr3 loss (light green) and not in the 3 tumours with chr3p loss (dark green), which supports the hypothesis that either the loss occurred 10 independent times or the loss occurred very early in a founding cell.

The copy number results provided further evidence that the tumours were independent because tumours within a patient had different sCNVs (Fig. 4a). Some chromosome regions, such as 3p and 5q, frequently have sCNVs in ccRCC. Tumours from the same patient showed distinct break points on both 3p and 5q indicating that these sCNVs arose independently.

Patients M28purple, M45orange and F58blue had both matched normal blood and adjacent kidney tissue, so copy number variants were called using both. As expected, the patterns were nearly identical, although the normal sample with the highest sequencing quality produced the least fragmented copy number calls. We also compared the blood and normal kidney tissue to each other and found no broad copy number variants, as expected.

An intriguing copy number variant pattern was observed in tumours from patient F28green. Out of the 13 tumours from this patient that were sequenced, 10 had a complete loss of one copy of chromosome 3 (Fig. 4a). The 3p arm of chromosome 3 is lost in >90% of ccRCC cases; however, the complete loss of chromosome 3 is a much rarer event, <10% in The Cancer Genome Atlas ccRCC cases^5^. It is highly unlikely that 10 out of 13 of her tumours lost all of chromosome 3 by chance if the likelihood of losing the complete chromosome in each given tumour is <10% (*P*<3 × 10^−8^, binomial). A close inspection of the sSNVs in patient F28green argues that the 10 tumours do not share a common lineage (Fig. 4b). No single-nucleotide variants were called in the 10 tumours with complete chromosome 3 loss and not in the 3 tumours with only chromosome 3p loss, which supports the hypothesis that either the loss of a complete copy of chromosome 3 occurred 10 independent times or the loss occurred very early in a founding cell.

## Discussion

VHL disease is one of only a few human conditions that results in the independent development of many tumours. Consistent with earlier studies using sCNVs (ref. 13) and sSNVs in coding exons^12^, we confirm that tumours from the same patient have distinct somatic variant sets. Of the ∼100,000 mutations we identified, the vast majority of somatic variants we observed were found in only one tumour in a patient. In <10% of the variants, reads matching the variant were found in more than one tumour per patient; and, in most of those cases, very few reads were observed in the other tumour(s). There are a number of reasons a read from a variant in one tumour could be found in another tumour. Hypothetically, a cell or DNA from one tumour could have migrated to the other tumour through the blood vessels, as ccRCC tumours are highly vascularized. Other explanations include tumour adjacency, cross-contamination during surgery or sample preparation, highly mutable DNA loci or simply sequencing error. In the small number of variants with significant read counts in several tumours, sequencing of both blood and normal kidney revealed that few appeared to be common only to the tumours. They could be common to the progenitor of the entire kidney, or more likely, they could have been lost (or not observed for technical reasons) in the progenitors of the myeloid system.

Cancer studies in humans are complicated by the fact that we have a wide diversity of genetic backgrounds and environments. To account for many confounding factors, often sample sizes in the thousands are required to even begin to stratify patients into groups. Population sizes in the millions may be necessary to adequately power statistical models that include genetic and environmental factors as well as their interactions. When studying cancer development in animal models, genetic background and environment can be held largely constant or varied in controlled experiments. By studying tumours from the same patient, we were able to observe how independent human cancers develop with genetic background and environment held constant. This allows us to estimate the importance of stochastic processes versus patient-specific factors. For example, one patient-specific factor which could influence tumour development is which germline VHL mutation is found in the patient. Previous studies have shown that how VHL is mutated in the germline can affect phenotype, such as age at first manifestation and risk of pheochromocytoma and retinal angiomas^25^,^26^. By extension, it is feasible that the type of VHL mutation could affect tumour development.

Our results show that in addition to the stochastic processes that drive tumour development, the combination of genetic background and environment also influences the types of mutations present in cancer. While independent tumours from the same patient have very different somatic variant sets, they share commonalities, such as the types of variants that are found. Somatic single-nucleotide substitution rates were significantly different between patients, and trinucleotide mutation context showed patient-specific patterns.

We also observed an example of a striking patient-specific bias in a chromosome-level copy number aberration. F28green is a young patient with a strong ccRCC phenotype that leads to the development of many tumours. This is unusual because clinical experience from treating patients with complete deletion germline variants of *VHL* usually finds a mild ccRCC phenotype^27^. This is thought to be due to the concurrent loss of neighbouring genes, such as *BRK1*, leading to a reduction in cell fitness when chromosome 3p is lost^27^. Patient F28green's germline deletion does not affect *BRK1*, potentially explaining her aggressive ccRCC phenotype. In the 13 tumours that we sequenced from F28green, 10 had completely lost the other copy of chromosome 3. Although most ccRCC cases lose chromosome 3p, the loss of all of chromosome 3 happens in fewer than 10% of cases. This leads us to conclude that either the complete loss of chromosome 3 occurred during kidney development or the genetic or environmental background of patient F28green increased the likelihood that all of chromosome 3 is lost during tumuorigenesis instead of just losing chromosome 3p.

The previous work in exome sequencing from VHL syndrome patients argued for functionally convergent evolution in a single patient for mutations that activate the PI3K/AKT/mTOR pathway^12^. We extend this observation by showing biases in both copy number as well as mutation spectra between individuals. We do not know if the biases in mutation spectra are caused by genetic or environmental constraints. We do believe that the whole-chromosome-3 loss phenotype is constrained, although we do not know if the differences between whole-chromosome-3 loss and retention of 3q are functional or if they represent evolutionary solutions that are more easily achieved but effectively equivalent.

The presence of additional patient-specific factors that drive or enhance tumuorigenesis in addition to the germline mutation of the *VHL* gene could also help explain the reasons for familial variability in clinical presentation. In a given family, not all affected individuals present with same severity of disease. Although this could be due to differing environmental factors, we suggest that the co-inheritance of additional genetic traits could affect symptomatic presentation. In some sense there is no distinction between somatic lesions that constrain tumour development as described in Fisher *et al*.^12^ and inherited lesions that constrain development as we describe here; however, we believe them to be worth considering independently as the constraints that arise from inherited variation are observable from the host (that is, in principle as early as the time of birth) while the constraints that arise in the development of a tumour can only be assayed once the tumour itself can be observed.

The ability to distinguish between genetic and environmental constraints is a challenging task in this model and in human tumours in general. One feasible future direction is to sequence multiple tumours from multiple surgeries from these patients, particularly in situations when the patient's environment changed, such as cessation of smoking or medication changes. Changes in mutation patterns would likely be attributed to environmental changes as genetic background stays constant. This study selected tumours from a single surgery to reduce the impact of time- and surgery-related factors; however, as the cost of tumour sequencing continues to fall, it will become possible to gather data like these on more patients, more tumours per patient, more surgeries per patient and more patients per family, allowing us to find additional within-patient patterns and make associations to environmental and genetic factors. Once patient-specific patterns are identified, we can begin to study the biological processes that cause the patterns to occur. Cancer susceptibility genes, environmental influences or an interaction of the two may initiate these processes. An understanding of how these patterns develop may aid in the prevention of human cancer and may help determine an individual's risk for developing cancer.

## Methods

### Tumour samples and DNA preparation

Patient phenotypes and other clinical data were obtained from the National Cancer Institute Clinical Research Information System or patient charts. This study was approved by the Institutional Review Board of the National Cancer Institute. All patients provided written informed consent.

DNA was extracted from frozen normal or tumour tissue with Maxwell 16 Tissue DNA purification kits (Promega) using the ‘tissue' programme. Blood DNA was extracted from EDTA-anticoagulated peripheral blood samples using Maxwell 16 Blood DNA purification kits (Promega) with the ‘buffy coat' programme. DNA concentrations were determined using a NanoDrop ND-1000 spectrophotometer (NanoDrop Technologies).

### Sequencing and alignment

The samples were sequenced using Illumina's Human Whole-Genome Sequencing service with a target coverage of 30x. Sequencing data files were shipped to Oregon Health & Science University on hard drives and analyses were performed on the Spellman lab compute cluster. HiSeq paired-end reads were aligned to the hg19 human reference genome using bwa-mem, an implementation of BWA v0.7.3 (ref. 28) that permits gapped alignments. Output sam files were converted to bam, sorted and indexed using samtools v0.1.17 (ref. 29). MarkDuplicates, part of Picard Tools v1.51 (ref. 30), was used to remove duplicate reads generated during the PCR amplification stage. Duplicate removal identifies all reads that have identical 5′ coordinates and keeps only the read pair with the highest base quality sums. After duplicate removal, fine-tuning of the alignment was performed using GATK v2.1 (ref. 31) as outlined in ref. 32 and summarized here: local positions to target for realignment were called using RealignerTargetCreator and then realigned using IndelRealigner. Quality scores were then recalibrated using BaseRecalibrator and PrintReads, which bins reads based on the original quality score, the dinucleotide and the position in the read.

### Variant calling and filtering

After creating high-quality alignments for each tumour and normal sample, somatic single-nucleotide and copy number variants were called by comparing the tumour samples to their matched normal(s). sCNVs were called using BIC-seq v1.1.2 (ref. 33) and sSNVs were called using MuTect v1.1.4 (ref. 34). In the patients with both matched blood and normal kidney tissue samples, we also called variants in blood versus tissue and tissue versus blood comparisons, with the expectation that most of the variants found in those comparisons would be false positives.

MuTect has high sensitivity and calls many variants even in regions of lower coverage. To reduce false positives, we performed three filtering steps: sample-level, dataset-level and pipeline-level. In the sample-level filtering, which considered each sample independently, called sSNVs were discarded if they had fewer than 14 reads in the tumour, fewer than 10 reads in the normal, <10% variant reads in the tumour or >2% variant reads in the normal. They were also discarded if they were suspected to be a single-nucleotide polymorphism (SNP). Our in-house SNP database includes all SNPs in dbSNP v134 (ref. 35) and those found by the NHLBI Exome Sequencing Project (downloaded 17Dec2012) (refs 36, 37) with the exception of the cancer-related variants found in COSMIC v60 (ref. 38).

The dataset-level filtering step took into consideration variant read frequencies across samples. By counting the reads that match all variants in all samples, we can identify and discard variants that are likely to be high sequencing error sites or common germline variants not found in the SNP database. Candidate sSNVs were discarded if unstable alignments (appearing as insertions and deletions) prevented them from being reliably quantified in the majority of samples. They were also discarded if reads matching the variant were seen in >10% of reads in another patient's sample but were not called by MuTect. Lastly, to remove variants that had a low read frequency in many samples, the Binomial distribution was used to determine if the number of reads matching a called variant exceeded the background rate, which was estimated using the proportion of reads matching that variant in samples from the other patients. The variant was discarded if the binomial *P* value exceeded 1 × 10^−8^. The combination of these dataset-level filtering steps is highly effective at removing false-positive whole-genome sequence sSNV calls while retaining true positives. We determined this two ways using a renal cell carcinoma data set from The Cancer Genome Atlas by comparing pre- and post-filtering MuTect whole-genome calls to: (1) The higher-coverage higher-confidence whole-exome calls from the same samples and (2) The pre- and post-filtering whole-genome calls using a secondary sSNV caller, Strelka.

The final filtering step, pipeline-level filtering, took the intersection of calls made by the current version of the pipeline and the previous version of the pipeline, which utilized bwa-aln v0.5.9, GATK v1.6 and MuTect v1.0.2. This further reduced false positives by eliminating questionable sSNV calls with borderline variant read frequencies from regions with unstable alignments.

### Tumour purity estimation

Tumour purity (Fig. 1 and Supplementary Data 1) was estimated by binning the filtered variants from each tumour into 20 bins based on their variant allele frequency (variant read counts/total read counts in tumour). The average of the variant allele frequencies in the bin containing the most variants was multiplied by two to produce an estimate of tumour purity. This approach reduces the contributions from potential subclonal variants or errors in the purity estimate. This approach for estimating purity is appropriate to use in tumours that are almost exclusively diploid, as ccRCC tumours are^39^. Purity estimates from this method were highly correlated with estimates from ABSOLUTE (ref. 39) in the 32 tumours where ABSOLUTE was able to fit a suitable model (y=0.9357x+0.0663, *R*^2^=0.90086, *P*<0.00001; Supplementary Data 1).

## 

## Additional information

**Accession codes:** The whole-genome sequencing data have been deposited in the dbGaP database under accession code phs001107.v1.p1.

**How to cite this article:** Fei, S. S. *et al*. Patient-specific factors influence somatic variation patterns in von Hippel–Lindau disease renal tumours. *Nat. Commun.* 7:11588 doi: 10.1038/ncomms11588 (2016).

## Supplementary Material

Supplementary Data 1 Sample information and each tumor vs. normal, and normal vs. normal comparison that was performed. Information includes coverage, sSNV counts, estimated tumor purity, tumor grade and size.

## Figures and Tables

**Table 1:** Patients selected for sequencing and number of tumours sequenced.

Patient ID	Sex	Age at surgery	Germline VHL mutation	Number of surgeries	Smoker	BMI	Tumours sequenced	Adjacent normal tissue
M45orange	Male	45	delA, fsArg176	2nd R (1 prior L, 1 subs L)	Former- 12 pack years	24.0	6 (5*)	Yes
M28purple	Male	28	Asn78Ser	1st R (no L)	Yes- 1 pack per day	27.4	6	Yes
M22red	Male	22	Pro86Arg	1st R (2 subs R, no L)	Former- <10 pack years	33.4	6 (5*)	No
F60yellow	Female	60	Trp117Cys	3rd R (prior L radical)	Never	24.7	6	No
F58blue	Female	58	delC, fsThr105	1st R (1 prior L plus L total)	No	22.6	5	Yes
F28green	Female	28	Complete deletion	2nd R (1 prior L)	No>Yes	21.6	13	No

^*^BMI, body mass index.

^*^One sample from patient M22red and one sample from patient M45orange had few variants and clustered with the normal samples. Further inspection showed these samples were cyst-like rather than tumours, so they were excluded from further analysis.
